# Variation in angler distribution and catch rates of stocked rainbow trout in a small reservoir

**DOI:** 10.1371/journal.pone.0190745

**Published:** 2018-01-11

**Authors:** Brian S. Harmon, Dustin R. Martin, Christopher J. Chizinski, Kevin L. Pope

**Affiliations:** 1 Nebraska Cooperative Fish and Wildlife Research Unit, and School of Natural Resources, University of Nebraska–Lincoln, Lincoln, NE, United States of America; 2 School of Natural Resources, University of Nebraska–Lincoln, Lincoln, NE, United States of America; 3 U.S. Geological Survey–Cooperative Fish and Wildlife Research Unit, and School of Natural Resources, University of Nebraska–Lincoln, Lincoln, NE, United States of America; Edith Cowan University, AUSTRALIA

## Abstract

We investigated the spatial and temporal relationship of catch rates and angler party location for two days following a publicly announced put-and-take stocking of rainbow trout (*Oncorhynchus mykiss*). Catch rates declined with time since stocking and distance from stocking. We hypothesized that opportunity for high catch rates would cause anglers to fish near the stocking location and disperse with time, however distance between angler parties and stocking was highly variable at any given time. Spatially explicit differences in catch rates can affect fishing quality. Further research could investigate the variation between angler distribution and fish distribution within a waterbody.

## Introduction

Recreational anglers and sportfish interact across a spatially and temporally dynamic, multi-scale landscape [1–3]. Catch rates are affected by the behavior and skill of anglers [4–5] as well as the behavior and catchability of fish [6]. Catch rates vary with space [7] and time [8], and also among anglers [9]. Put-and-take fisheries provide short-term opportunities for relatively high catch rates and are a method for recruiting and retaining anglers [10]. In publicly announced put-and-take stocking events, anglers often have detailed information regarding stocking location.

Where anglers choose to fish within put-and-take fisheries may affect the number of fish caught. Commercial fishermen were found to track overall abundance of fish [11–12], but did not conform to an ideal free distribution [13–14] even after accounting for differences in skill [15]. Similarly, anglers appear to mismatch the spatial distribution of fish [7]. In a put-and-take fishery, where the initial stocked fish distribution is determined by stocking locations, a spatial mismatch may lead to significant differences in catch rates.

We surveyed angler parties in Holmes Lake, an urban put-and-take fishery in Lincoln, NE, following a publicly announced stocking of Rainbow Trout, *Oncorhynchus mykiss*, by the Nebraska Game and Parks Commission (NGPC). We wanted to examine the effect of angling location (space and time) on catch rates of rainbow trout. We examined the distribution of angler parties and their catch rates of rainbow trout. We hypothesized that catch rates would decline with both distance and time since stocking. We expected that angler parties would congregate around the stocking location immediately following stocking and gradually disperse through time with a portion of angler parties continuing to fish near the stocking point to maximize catch rates.

## Materials and methods

### Study site

Holmes Lake is a 45-ha flood-control reservoir located in Lincoln, Nebraska, (40°46'48.0"N 96°37'48.0"W) consists of two basins connected by a channel roughly 60-m wide with a maximum depth of 5-m, and is open daily to the public from 0500 to 2300. Holmes Lake is stocked with catchable-size (>250-mm total length) rainbow trout during spring and fall by the NGPC to provide short-term angling opportunities in a system not suitable for a persistent rainbow trout population. We confined our study to the southern basin (9-ha, maximum depth of 3-m), bounded to the north by a passenger bridge that historically was netted to block rainbow trout dispersal, and bounded to the south by unfishable marshy habitat. We notified the Nebraska Game and Parks Commission of the survey, but permission to survey at Holmes Lake was not necessary because the property is public land. This field study did not involve endangered or protected species. Few trout were likely to have left the study site [16] and few anglers were present outside of this boundary. Additionally, no interviewed anglers outside our study site caught rainbow trout.

### Creel surveys

On October 15, 2012, at 1400, 3,488 rainbow trout were stocked by the NGPC at the southern boat ramp and fishing began immediately. We assumed that all trout belonged to this cohort because summer temperatures during 2012 were fatal for any un-captured trout from previous stocking events. We conducted a roving creel survey [17] of bank anglers (complete and incomplete trips) from 1100 to 2200 hours, October 15, and from 0600 to 2200 hours, October 16 (S1 Table). Angler parties were approached at their fishing location by a creel survey technician who intercepted angler parties during their fishing trip at their fishing location. This study was approved by the University of Nebraska-Lincoln Institutional Review Board (protocol #2008129518 EX). Anglers were approached and verbally asked for consent to participate in a survey because written consent was deemed a barrier to high participation rates. Consent and refusal were recorded on a survey (S1 Text). This methodology was reviewed and approved by the University of Nebraska-Lincoln Institutional Review Board. Anglers were interviewed at the party level; in each interview one angler answered for the party. We interviewed parties no more than once per hour to collect their target species, and hourly catch and harvest of trout. Catch rates (catch of trout during the previous hour) were used for analysis. We recorded the timestamp of the interview as the number of hours since the stocking occurred. We excluded parties that self-identified as targeting anything other than "trout" or "anything" from the analysis of spatial catch rates.

Each hour we recorded the number, location, and size of angler parties in the southern basin on a detailed map of Holmes Lake (S2 Table). The straight-line distance from all observed and interviewed angler parties to stocking location was calculated for each hour. We compiled and analyzed data with ArcGIS version 10.2.2 and program R version 3.2.3 [18]. Anglers fishing from boats (N = 9) were excluded from the analyses because they were too few (9 of 204) to be considered separately.

### Modeling

We analyzed catch rates (dependent variable) as a function of two independent variables distance from stocking location(continuous) and time since stocking (categorical: two groups per day for two days) with generalized linear models (GLM's) with Poisson and negative binomial links (S2 Text). We assessed spatial autocorrelation with a Moran's I test with R package ape [19] with the raw data and of the residuals from the top performing model. With Time was categorized because a continuous time variable would estimate catch rates over a substantial amount of time when no anglers fished (i.e. when the park was closed). We grouped angler interviews into two groups per day to account for within and between day variance. Angler interviews were grouped on October 15 between 1500 and 1759 hours and between 1800 and 2200 hours. Interviews from October 16 were grouped between 0600 to 1459 hours and between 1500 to 2200 hours. We included an offset of the log-transformed party size to account for the effect of party size on catch rates. We tested four models: two negative binomial models and two Poisson models that differed by whether a zero-inflation term was included. Prior examination indicated a majority of anglers caught no trout within the previous hour. We ran the models with R package glmmADMB [20] and selected our model with Akaike information criterion (ΔAIC). We used the predict function in program R to build prediction rasters.

## Results

### Fishery description

We recorded 195 angler parties during hourly counts and collected 143 interviews from angler parties in the southern basin of Holmes. Mean party size was 1.65±0.07 anglers. Interviewed anglers caught 74 trout and harvested 63 of those trout. One hundred twenty-two angler parties self-reported as primarily seeking rainbow trout, of which 71 interviews were with previously interviewed parties. Eleven parties reported primarily seeking "anything". Ten parties sought other species and were subsequently excluded from analyses.

**Fig 1:**
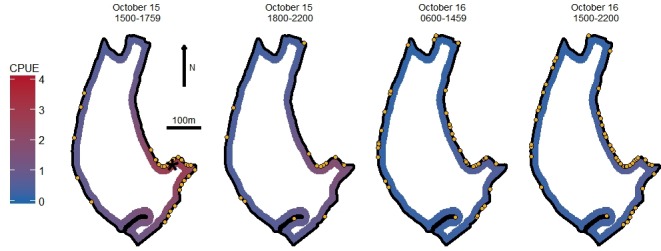
Model predictions of catch per hour. Modeled outputs from the top candidate model mapped onto the southern basin of Holmes Lake. Points represent the location of angler parties counted during each time period. The asterisk in the first panel represents the stocking location of rainbow trout. Original shapefile provided by Nebraska Game and Parks Commission.

Catch rates declined with both time since stocking and distance from stocking location (Fig 1). Mean distance ± SE from angler parties to stocking location increased with time from 91m± 99 in the time period immediately following stocking to 144m± 114 by the end of the second day. Although distance from stocking site increased with time, time explained little of the total variation (ANOVA, F_3,186_ = 2.62, P = 0.053, *r*^2^ = 0.04).

### Modeling

We did not detect spatial autocorrelation in our catch rate data (Moran's I = 0.008, P = 0.50). In our analysis of catch rates, two candidate models had comparable AIC values (Table 1). The first candidate model was a zero-inflated Poisson model and the second candidate model was a zero-inflated negative binomial model. All other model ΔAIC values were > 4. We elected to use parameters from the first candidate model to predict catch rates (Fig 1) because the model contained fewer parameters (Occam’s razor[21]). We did not detect a significant difference in catch rates during the first day between periods, but did detect a significant decline during the second day. [Table 1 near here] We did not detect spatial autocorrelation in the residuals from the top model (Moran's I = 0.03, P = 0.13).

**Table 1:** Akaike information criterion (AIC) results of candidate models comparing the effect of time and distance (independent variables) on catch rates (dependent variable).

Family	Zero inflation	ΔAIC	Weight
Poisson	Yes	0	0.7
Negative binomial	Yes	1.9	0.3
Poisson	No	10.4	<0.1
Negative binomial	No	24.8	<0.1

## Discussion

We expected angler parties to initially congregate near the rainbow trout stocking location when fish were stocked and then expand across the reservoir through time. However, angler-party mean distance was highly variable, and time since stocking explained little of the total variation in catch rates. A portion of angler parties fished in areas too distant to expect high catch rates of rainbow trout during the study [22–23] because stocked fish distributed is typically a leptokurtic function of distance from a stocking site [16] although some studies of lentic trout described extensive movement [23–25]. At greater stocking densities trout are more likely to school, [26] which may help explain the dense concentrations of trout caught near the stocking point.

Managers typically use put-and-take fisheries as a tool to provide anglers with increased catch rates and as a method for recruiting and retaining anglers [10]. However the dramatic decrease in catch rates through the two-day period indicates the greatest boost in catch rates from put-and-take fisheries is short term. Relatively few fish (N = 74) were captured compared to the number stocked (N = 3488), and most angler parties interviewed (N = 92) had not caught a trout during the previous hour. Although we did not address other factors influencing site choice, anglers may have optimized for factors other than catch rates when selecting a site. At larger spatial scales infrastructure affects angler site choice [27]. In addition to infrastructure, anglers did not fish in isolation from one another, but instead were able to watch the outcomes of other anglers’ decisions. For example, a fishing pier near (<100 m) from the stocking location was heavily fished likely because anglers knew that it was near the stocking location but also because they watched anglers catch trout from the pier. Anglers may also have selected for other infrastructure or social situations that led to significantly reduced catch rates.

Although catch-rates are not the only factor influencing angler satisfaction [28], improved catch rates may improve angler satisfaction. Locations thought to attract angler use within a waterbody could be matched with stocking locations to improve angler catch rates, and meet local objectives of put-and-take fisheries. In systems where stocking can provide ecological benefit, managers may want to consider stock in locations where fish are less likely to be immediately caught to optimize for ecosystem services other than recreation [29]. Future work could consider the effect of the spatial mismatch between anglers and fish in recreational fisheries.

## Supporting information

S1 Table Angler responses to survey. (CSV)

S2 Table Angler locations. (CSV)

S1 Text Copy of angler creel survey. (PDF)

S2 Text Program R script used for data analysis. (TXT)
