# Supplementary material for: Variation in angler distribution and catch rates of stocked rainbow trout in a small reservoir
Source: PLoS One. 2018 Jan 11;13(1):e0190745. doi: 10.1371/journal.pone.0190745 (PMC5764306; doi:10.1371/journal.pone.0190745)
Supplement: S1 Text — (PDF) [file pone.0190745.s003.pdf]

What time did you begin fishing today?

What is the primary species of fish you are seeking?

Have you been interviewed earlier today?

Were you aware of the trout stocking before arriving today?

Was the trout stocking the primary purpose of your trip?

How many rainbow trout have you caught in the last hour?

How many rainbow trout have you harvested in the last hour?
